# Clinical relevance of circulating cell-free microRNAs in ovarian cancer

**DOI:** 10.1186/s12943-016-0536-0

**Published:** 2016-06-24

**Authors:** Koji Nakamura, Kenjiro Sawada, Akihiko Yoshimura, Yasuto Kinose, Erika Nakatsuka, Tadashi Kimura

**Affiliations:** Departments of Obstetrics and Gynecology, Osaka University Graduate School of Medicine, 2-2 Yamadaoka, Suita, Osaka 565-0871 Japan

**Keywords:** Circulating miRNA, Ovarian cancer, Serum, Plasma, Early detection

## Abstract

Ovarian cancer is the leading cause of death among gynecologic malignancies. Since ovarian cancer develops asymptomatically, it is often diagnosed at an advanced and incurable stage. Despite many years of research, there is still a lack of reliable diagnostic markers and methods for early detection and screening. Recently, it was discovered that cell-free microRNAs (miRNAs) circulate in the body fluids of healthy and diseased patients, suggesting that they may serve as a novel diagnostic marker. This review summarizes the current knowledge regarding the potential clinical relevance of circulating cell-free miRNA for ovarian cancer diagnosis, prognosis, and therapeutics. Despite the high levels of ribonucleases in many types of body fluids, most of the circulating miRNAs are packaged in microvesicles, exosomes, or apoptotic bodies, are binding to RNA-binding protein such as argonaute 2 or lipoprotein complexes, and are thus highly stable. Cell-free miRNA signatures are known to be parallel to those from the originating tumor cells, indicating that circulating miRNA profiles accurately reflect the tumor profiles. Since it is well established that the dysregulation of miRNAs is involved in the tumorigenesis of ovarian cancer, cell-free miRNAs circulating in body fluids such as serum, plasma, whole blood, and urine may reflect not only the existence of ovarian cancer but also tumor histology, stage, and prognoses of the patients. Several groups have successfully demonstrated that serum or plasma miRNAs are able to discriminate patients with ovarian cancer patients from healthy controls, suggesting that the addition of these miRNAs to current testing regimens may improve diagnosis accuracies for ovarian cancer. Furthermore, recent studies have revealed that changes in levels of cell-free circulating miRNAs are associated with the condition of cancer patients. Discrepancies between the results across studies due to the lack of an established endogenous miRNA control to normalize for circulating miRNA levels, as well as differing extraction and quantification methods, are the pitfalls to be resolved before clinical application. There is still a long way, however, before this can be achieved, and further evidence would make it possible to apply circulating cell-free miRNAs not only as biomarkers but also as potential therapeutic targets for ovarian cancer in the future.

## Background

Ovarian cancer is the leading cause of gynecological cancer-associated deaths in developed countries. In the United States, ovarian cancer is the fifth leading cause of cancer death in females, with an estimated incidence of 14,180 deaths in 2015 [1]. High-grade serous ovarian cancer (HGSOC) accounts for 70–80 % of ovarian cancer deaths. While this disease is termed an ovarian cancer, a wide range of studies have suggested that secretory epithelial cells of the distal fallopian tube are the likely progenitors of a substantial proportion of HGSOCs, although some HGSOCs arise without fallopian tube [2]. While the survival rates of patients diagnosed with early stage ovarian cancer are high, most cases are diagnosed at a late stage with peritoneal dissemination. If diagnosed at an early stage, the 5-year survival rate exceeds 90 %. However, in patients with stage III or IV ovarian cancer, despite comprehensive treatments by aggressive cytoreductive surgery and chemotherapy with platinum- and taxane-based drugs, the 5-year survival rate remains at a dismal 30 % [1]. This high mortality rate of ovarian cancer is associated with the difficulties of early detection, because it is usually asymptomatic until late stage. Efficient early detection procedures have yet to be established. Although pelvic examination, transvaginal ultrasonography, and serum carbohydrate antigen 125 (CA125) are usually performed as routine diagnostic procedures for ovarian cancer, their diagnostic values are limited due to lack of the sensitivity and specificity [3]. For example, CA125 is only elevated in 50–60 % patients with stage I and II ovarian cancer [4]. In the retrospective study with 751 females, the sensitivity of CA125 for ovarian cancer at all stages was 88.6 %, but with a specificity of only 72.0 % [4]. In the recent study with 118 patients with ovarian cancer, 84 with benign disease, and 61 healthy females, the sensitivity and specificity of CA125 were 77.4 and 70.8 %, respectively [5]. Therefore, development of novel approaches to effectively detect ovarian cancer at an early, curable stage is urgently required. Another cause of this high mortality rate is the resistance to chemotherapy, especially in recurrent cases. Although ovarian cancer is highly responsive to the initial treatment by platinum- and taxane-based chemotherapies, subsequent relapses and repeated treatments using these cytotoxic chemotherapies eventually result in acquired resistance to the treatments. Therefore, most patients who experience cancer relapse eventually succumb to the disease [6]. In the last decade, a variety of targeted therapies have been developed to target the cancer-specific genes and proteins or the tumor microenvironment that contributes to cancer growth and maintenance. Among the new drugs studied for ovarian cancer, bevacizumab, an anti-vascular endothelial growth factor (VEGF) antibody, has shown promising activities in combination with standard chemotherapies in large Phase III trials [7, 8]. However, no significant benefits with respect to overall survival have been reported so far. Poly ADP ribose polymerase (PARP) inhibitors, which lead to formation of extensive double-stranded DNA breaks that cannot be accurately repaired in tumors, have shown potential for improved survival. Olaparib, a potent oral PARP inhibitor, significantly improved progression-free survival in patients with platinum-sensitive, relapsed, high-grade serous ovarian cancer; however, interim analysis showed no overall survival benefit [9]. Therefore, definitive treatments that substantially extend overall patient survival have yet to be established [10]. This is partly due to the lack of methods to discriminate between patients who will or will not benefit from the specific molecular targeted treatments. Thus, identification of useful clinical biomarkers to predict possible resistance to each treatment and prognoses of cancer patients would greatly benefit the management of ovarian cancer treatment [11]. Accumulating evidence has revealed that microRNAs (miRNA or miR) are extensively involved in cancer progression and suppression by regulating thousands of cancer-associated genes [12]. miRNAs can stably exist not only in cytoplasm, but also in various types of body fluids. Circulating cell-free miRNAs have been shown to have the potential to enable earlier cancer diagnosis and to predict prognosis and response to therapy [13]. This review summarizes the cumulative efforts in the field of circulating miRNAs focusing on their potential as novel biomarkers in ovarian cancer.

## miRNAs and ovarian cancer

miRNAs are endogenously expressed, single-stranded non-coding RNAs that are typically 19–25 nucleotides in length. They are initially generated as long primary miRNAs (pri-miRNAs) by RNA II polymerase-mediated transcription in the cell nucleus. The primary miRNAs are cleaved into approximately 70 nucleotide-long precursor miRNAs, which are subsequently transported into the cytoplasm and further cleaved into mature miRNAs [14]. miRNAs predominantly act as transcriptional repressors by binding the 3’ untranslated region of their target messenger RNAs. It is well established that miRNAs are key regulators of post-transcriptional gene expression [14, 15]. In mammals, miRNAs are estimated to control the activities of more than 50 % of all protein-coding genes [16] and have been shown to be involved in the regulation of almost all cellular processes [17]. Drosha and Dicer are essential enzymes for the biogenesis of miRNA. Drosha, an RNase III enzyme, cleaves the pri-miRNA and releases a hairpin-structured pre-miRNA in the nucleus. After the pre-miRNA is exported into the cytoplasm, Dicer, another RNase III enzyme, cleaves the pre-miRNA and releases the miRNA duplexes. In ovarian cancer patients, low Dicer expression is significantly correlated with advanced stage ovarian cancer, and low Drosha expression with suboptimal surgery [18], suggesting that impaired processing of miRNAs by Dicer and Drosha is involved in the tumorigenesis of ovarian cancer, and it leads to poor clinical outcomes [19]. Many studies have shown that dysregulation of miRNAs is involved in a variety of human diseases including ovarian cancer [19, 20]. The Cancer Genome Atlas (TCGA) project analyzed mRNA expression, miRNA expression, promoter methylation, and DNA copy number in a total of 489 HGSOCs and showed that ovarian cancers could be separated into 3 miRNA subtypes [21]. Among these, a robust integrated mesenchymal subtype was associated with poor survival, and eight key miRNAs (miR-25, miR-29c, miR-101, miR-128, miR-141, miR-182, miR-200a, and miR-506) were identified [22]. Mining the TCGA data, Miles et al. identified 17 miRNAs that were dysregulated in HGSOCs in comparison with normal ovary samples, including eight upregulated miRNAs (miR-183-3P, miR-15b-3p, miR-15b, miR-590-5p, miR-18a, miR-16, miR-96, and miR-18b) and nine downregulated miRNAs (miR-140-3p, miR-145-3p, miR-143-5p, miR-34b-5p, miR-145, miR-139-5p, miR-34c-3p, miR-133a, and miR-34c-5p) [23, 24]. Emerging evidence has revealed the role of microRNAs in ovarian cancer as oncogenes or tumor suppressor genes. For instance, Ohyagi-Hara et al. identified that miR-92a inhibits peritoneal dissemination of ovarian cancer cells by inhibiting integrin α5 expression, which had previously been shown as a key molecule in peritoneal dissemination [25, 26]. Kinose et al. demonstrated that miR-199a-3p is downregulated in high-grade serous ovarian carcinomas, and the loss of miR-199a-3p is involved in ovarian carcinogenesis and peritoneal dissemination by causing the upregulation of c-Met, a receptor against hepatocyte growth factor [27]. Davidson et al. and Zhang et al. summarized the roles of miRNAs in ovarian cancer in their review papers [24, 28].

## miRNAs in circulation

**Fig. 1:**
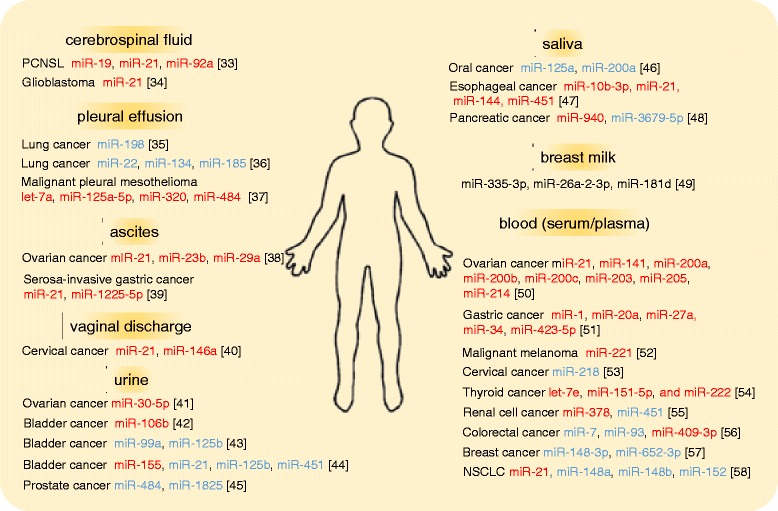
miRNAs in various human body fluids. miRNAs can be detected in various human body fluids. Circulating miRNAs reported as potential non-invasive diagnostic markers for many types of cancers are shown. miRNAs which were reported to be upregulated in cancer patients compared to controls (healthy or benign) are shown in *red*, and those downregulated in cancer patients are shown in *blue*. In [39], miR-21 and miR-1225-5p were overexpressed in ascites from serosa-invasive gastric cancer patients compared with non-invasive gastric cancer patients. In breast milk, 3 most abundant miRNAs are listed in the figure. PCNSL: primary central nervous system lymphoma; NSCLC: non-small cell lung cancer

**Fig. 2:**
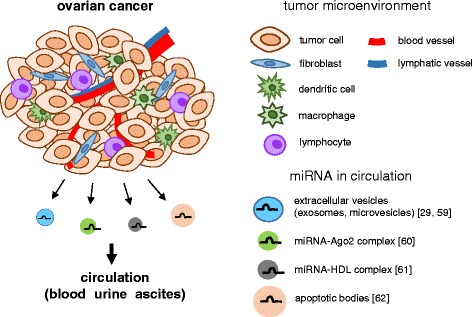
Circulating miRNAs derived from ovarian cancer. Ovarian cancer-associated miRNAs are reported to be detected from blood (serum/plasma), urine, or ascites. MiRNAs in circulation display remarkable stability. They are encapsulated by membrane-enclosed vesicles such as exosomes and microvesicles, or bound to carrier protein or lipids such as Argonaute2 (Ago2) and HDL. miRNAs are protected by these miRNA-carriers from RNase in circulation [29, 59–62]. HDL: high density lipoprotein

Since high levels of endogenous nucleases are present in body fluids, freely circulating RNAs were expected to be rapidly degraded. However, recent evidence has revealed that miRNAs are secreted from cells into circulation where they exist stably, and sometimes can be functionally transferred to recipient cells [29, 30]. Circulating miRNAs were first detected in the serum and plasma [31, 32] in 2008, and subsequently found in a variety of body fluids, such as saliva, urine, breast milk, and so on [33–58] (Fig. 1). One of the first studies measuring miRNA levels in the serum was reported by Mitchell et al. [31], who found that miRNAs are present in human plasma in a remarkably stable form that is protected from endogenous RNase activity. They could distinguish patients with prostate cancer from healthy subjects by measuring the serum levels of miR-141. In 2009, Park NJ, et al. reported that endogenous salivary miRNAs were degraded at much slow rates compared with exogenous miRNA, and that both miR-125a and miR-200a were present at significantly lower levels in the saliva of oral squamous cell carcinoma patients than in control subjects, indicating the presence of miRNA in saliva [46]. Currently, circulating miRNAs are known to be remarkably stable under harsh conditions, such as extreme pH values, high temperatures, and long-term storage [31, 32, 56]. Current studies have suggested that miRNAs are released from cells into circulation by using several packaging and transportation systems to avoid degradation (Fig. 2): (a) Active secretion: miRNAs are encapsulated in extracellular membrane vesicles such as exosomes (30–100 nm) or microvesicles (50–1000 nm) [29, 59]. They are also secreted via binding to miRNA-binding proteins, such as AGO2, or serum lipid carriers, such as HDL [60, 61]. (b) Passive leakage: miRNAs are involved in shedding vesicles, such as apoptotic bodies (−4000 nm), as a result of apoptotic death [62]. Previous studies have demonstrated that miRNA expression profiles are different depending on the physiological and pathological conditions [63]. In other words, some miRNAs are upregulated or downregulated specifically in certain cancer types. This stability and specificity make circulating miRNAs potential biomarkers of cancer diagnosis [13].

## Methodologies for the collection and analysis of circulating miRNAs

The expression profile of circulating miRNAs is highly affected by the methodologies of sample preparation [60, 64]. How to prepare samples adequately depends on the types of body fluids. In ovarian cancer research, samples were generally collected from blood, ascites, or urine. Blood samples were firstly processed into serum or plasma in most studies. For urine analyses, morning whole-stream urine samples were collected [41]. Ascites was collected for routine diagnostic purposes during gynecological surgeries or abdominocentesises. Thereafter, they were centrifuged to avoid contamination of cell fraction and stored in −80 °C [41, 65–80]. In exosomal miRNA research, exosomes were collected using an ultracentrifugation method or a commercial-based exosome isolation kit such as ExoQuick (SBI, Palo Alto, CA) [30, 50]. Thereafter, the extraction of RNA is usually performed by using commercially available techniques including phenol/guanidinium products, such as TRIzol (Life Technologies), and column-based extraction kits, such as mirVana (Life Technologies) and miRNeasy (QIAGEN).

In most studies, the extracted miRNAs are subjected to next-generation sequencing (NGS) or miRNA microarrays to obtain large-scale profiles of circulating miRNAs and determine candidate miRNAs for further quantification. Each method has certain advantages and limitations. NGS has a potential to identify novel miRNAs but is less cost-effective and less efficient compared with microarrays. Thereafter, the candidate miRNAs are generally subjected to further validation by quantitative reverse transcription polymerase chain reaction (qRT-PCR) in larger cohorts. Establishment of endogenous controls for miRNA normalization remains the bottleneck for the reliable quantification of miRNAs. While RNU-6B, RNU-48, and miR-16 are commonly used as endogenous controls, no definitive control gene has been established [13, 81]. Recent studies have suggested more reliable endogenous controls for the quantification of circulating miRNAs [82, 83]. Chen et al. reported that a combination of let-7d, let-7 g and let-7i serves as an endogenous control of serum miRNAs and it is superior to the commonly used reference genes [82]. Kok et al. suggested normalization panels for the better quantification of circulating microRNAs by RT-qPCR [83]. Further studies with a large number of samples would be needed to determine a suitable reference for reliable normalization of circulating miRNAs.

## Clinical relevance of cell-free miRNAs in ovarian cancer

Since 2008, numerous studies have demonstrated the clinical relevance of circulating miRNAs as diagnostic and prognostic biomarkers for a variety of cancer types, using blood plasma or serum. Cancer-associated circulating miRNA expression profiles were determined and have been shown to be related to tumor development, disease progression, and metastases. This section describes the potential roles of circulating miRNAs as novel biomarkers in regard to diagnosis and prognosis in ovarian cancer.

1. Diagnostic potential of circulating miRNAs in ovarian cancer
  Approximately 20 studies have been published on the diagnostic potential of circulating miRNAs in ovarian cancer. Taylor et al. first reported that eight exosomal miRNAs (miR-21, miR-141, miR-200a, miR-200b, miR-200c, miR-203, miR-205, and miR-214) from sera were elevated in ovarian cancer patients compared to benign controls, which had been reported to be overexpressed in ovarian cancer tissues. These miRNAs were overexpressed even in patients with early stages of ovarian cancer. The miRNA signatures from exosomes were parallel to those from the originating tumor cells, indicating that circulating miRNA profiles accurately reflect the tumor profiles [50]. Following this study, various reports have demonstrated the diagnostic potential of circulating miRNAs in body fluids such as serum, plasma, whole blood, and urine [41, 65–80] as summarized in Table 1. Hauser et al. investigated the miRNA expression pattern in relapsed ovarian cancer patients from whole blood samples containing cellular fraction by miRNA array. They reported that miR-30c-1-3p was upregulated, and miR-342-3p, miR-181a-3p, and miR-450b-5p were downregulated in patients with relapsed ovarian cancer when compared to healthy controls [66]. Kan et al. reported that the expression levels of four serum miRNAs including the miR-200 family (miR-182, miR-200a, −200b, and -200c) were significantly elevated in serous ovarian cancer patients compared with healthy volunteers. They identified a multivariate model combining miR-200b and miR-200c that has good predictive power to discriminate patients with serous ovarian cancer and healthy controls (AUC = 0.784), and this model may have utility as potential biomarkers of serous ovarian cancer [67]. Chung et al. analyzed an miRNA array using RNAs isolated from serum, tissue, and ascites from serous ovarian cancer patients and a healthy control, and identified five miRNAs (miR-132, miR-26a, let-7b, miR-145, and miR-143) as the most significantly downregulated miRNAs in the sera of ovarian cancer patients with respect to those of the control [68]. Zheng et al. evaluated plasma samples of 360 epithelial ovarian cancer patients and 200 healthy controls and found higher expression of plasma miR-205 and lower expression of let-7f in the ovarian cancer patients. Combination of miR-205 and let-7f together provided high diagnostic accuracy for epithelial ovarian cancer (AUC = 0.831 (95 % CI, 0.772 to 0.880; sensitivity = 62.4 %, specificity = 92.9 %), particularly in patients with stage I disease, indicating that these two miRNA signatures could be used as biomarkers of ovarian cancer detection, particularly for stage I disease [69]. Suryawanshi et al. focused on endometriosis-associated ovarian cancer (EAOC) (endometrioid adenocarcinoma or clear cell carcinoma) and revealed three plasma miRNAs, miR-16, miR-191, and miR-195, all upregulated in endometriosis, that could differentiate between healthy and endometriosis cases with 88 % sensitivity and 60 % specificity. They further showed that a combination of miR-16, miR-21, and miR-191 could differentiate between healthy and EAOC with 86 % sensitivity and 85 % specificity and that miR-21, miR-191, and miR-1975 together could distinguish between EAOC and serous ovarian cancer with 86 % sensitivity and 79 % specificity [70]. Zuberi et al. reported that serum miR-200a is significantly upregulated in mucinous adenocarcinomas compared with other types of histology in 70 epithelial ovarian cancer patients and at the highest AUC value, the sensitivity and specificity of this miRNA relative expression were 80.6 and 73.5 %, respectively [79]. Meng et al. revealed that serum levels of mR-25 and miR-93 were downregulated, while those of miR-7 and miR-429 were upregulated in 180 epithelial ovarian cancer patients compared with 66 healthy women. These four miRNA signatures discriminated ovarian cancer patients from healthy women with a high sensitivity and specificity: 93 and 92 %, respectively [75]. Zhou et al. reported diagnostic value of urinary miRNAs in ovarian cancer. They identified significant upregulation of miR-30a-5p in the urine sample of ovarian cancer patients when compared to healthy controls. They also identified that miR-30a-5p was concentrated in exosomes from ovarian cancer cell culture supernatant and urine from ovarian cancer patients, supporting a pathway for miR-30a-5p excretion into the urine [41]. Kapetanakis et al. showed that plasma miR-200b was significantly more abundant in patients with ovarian cancer than in those with benign tumors. They suggested the potential role of miR-200b as a complementary biomarker of CA125, because there was no significant correlation between the distributions of these markers [80].

**Table 1:** Circulating miRNAs as potential diagnostic biomarkers of ovarian cancer

Reference	Elevated miRNA	Decreased miRNA	Source	Tumor histology (n)		FIGO stage (n)		Control (n)		
				Serous	Others	I-II	III-IV	HC	Ben	Bor
[50], 2008	miR-21, miR-141, miR-200a, miR-200b, miR-200c, miR-203, miR-205, miR-214		Serum (exosome)	50		20	30	10	10	
[65], 2009	miR-21, miR92, miR-93, miR-126,miR-29a	miR-155, miR-127, miR-99b	Serum	17	11	10	18	15		
[66], 2010	miR-30c-1	miR-342-3p, miR-181a, miR-450-5p	Whole blood	21	3	Relapsed		15		
[67], 2012	miR-182, miR-200a, miR-200b, miR-200c		Serum	28		1	27	28		
[68], 2013		miR-132, miR-26a, let7-b, miR-145	Serum	18		3	14	12		
[69], 2013	miR-205	let-7f	Plasma	179	181	133	227	200		
[70], 2013	miR-16, miR-21, miR-191 (EAOC) miR-16, miR-191, miR-4284 (SOC)		Plasma	21	14	12	23	20	33	
[71], 2013	miR-21		Serum	68	26	32	62	40		
[72], 2013	miR-221		Serum	70	26	32	64	35		
[73], 2014	miR-191-5p, miR-206, miR-548a-3p, miR-320a, miR-574-3p, miR-590-5p, miR-34c-5p, miR-106b-5p	miR-19a-3p, miR-30a-5p, miR-645, miR-150-5p	Plasma	18	0	6	12	24		
[74], 2014	miR-1274a, miR-625-3p, miR-720	miR-106b, miR-126, miR-150, miR-17, miR-20a, miR-92a	Plasma	42		6	36	23	36	
[75], 2015	miR-7, miR-429	miR-25,miR-93	Serum	120	60	32	147	66		
[41], 2015	miR-30-5p		Urine	39		16	18	30	26	
[76], 2015	miR-141, miR-200c		Serum	16	58	54	20	50		19
[77], 2015		miR-145	Serum	18	64	31	63	135		
[78], 2015		let-7i-5p, miR-122, miR-152, miR-25-3p	Serum/plasma	20		6	19		25	
[79], 2015	miR-200a, miR-200b, miR-200c		Serum	70		33	37	70		
[78], 2015	miR-200b		Plasma	51		6	45	25	25	

FIGO: International Federation of Gynecology and Obstetrics, EAOC; endometriosis associated ovarian carcinoma, SOC; serous ovarian carcinoma, HC; healthy control, Ben; Benign control, Bor; borderline tumor
2. Potential of circulating miRNAs in ovarian cancer as prognostic biomarkers
  Current management of ovarian cancer relies on clinicopathological factors including tumor histology and stage. Recent studies have revealed that changes in the levels of circulating miRNAs are associated with the prognosis of ovarian cancer patients [38, 69, 71, 72, 74–77, 80], as summarized in Table 2. Zheng et al. demonstrated that lower plasma let-7f expression was significantly correlated with poor progression-free survival (PFS) in 360 ovarian cancer patients, particularly in stage III and IV cases. Cox regression analysis revealed that plasma let-7f was an independent prognostic indicator of ovarian cancer in PFS [69]. Zhao et al. investigated the serum level of miR-21 in a total of 94 epithelial ovarian cancer patients. They showed that increased serum miR-21 expression was associated with advanced FIGO stage, high tumor grade, and shortened overall survival (OS), and that a high level of serum miR-21 expression was an unfavorable prognostic factor independent of other clinicopathological factors [71]. Vaksman et al. studied miRNA expression in effusion-derived exosomes. Exosomes were collected from 66 malignant peritoneal and 20 pleural effusions obtained from a total of 86 ovarian cancer patients with advanced stages. They demonstrated that high levels of miR-21, miR-23b, and miR-29a were associated with poor PFS in univariate analyses. In addition, high expression of miR-21 was correlated with poor OS in Cox regression analysis [38]. Gao et al. investigated the diagnostic and prognostic potential of serum miR-200c and miR-141, which are members of the miR-200 family, in 74 ovarian cancer patients. Both miRNAs were significantly overexpressed in ovarian cancer patients compared to 50 healthy control. Patients with high miR-200c levels achieved a significantly higher 2-year survival rate, while the low miR-141 group showed a significantly higher survival rate [76].

**Table 2:** Circulating miRNAs as potential prognostic predictors of ovarian cancer

Reference	miRNAs associated with poor prognosis		Endpoint	Source	Patients (n)	Histology (n)		FIGO stage (n)	
	Increased miRNAs	Decreased miRNAs				Serous	Others	I-II	III-IV
[69], 2013		let-7f	PFS	Plasma	360	179	181	133	227
[71], 2013	miR-21		OS	Serum	94	68	26	32	62
[72], 2013	miR-221		OS	Serum	96	70	26	32	64
[74], 2014		miR-1290	OS	Plasma	26	26	0	6	36
[38], 2014	miR-21,miR-23b, miR-29a (PFS), miR-21(OS)		PFS/OS	Effusion (exosome)	86	76	10	0	86
[75], 2015	miR-429		OS	Serum	180	180		32	147
[76], 2015	miR-141	miR-200c	OS	Serum	74	16	58	54	20
[77], 2015		miR-145	OS	Serum	82	18	64	31	53
[80], 2015	miR-200b		PFS	Plasma	33	33		Unknown	

FIGO: International Federation of Obstetrics and Gynecology, PFS: progression free survival, OS: overall survival

Several research groups have studied the ability of miRNAs to predict the sensitivity to treatment. Kapetanakis et al. demonstrated the pre/post-treatment variations of plasma miR-200b in ovarian cancer as a potential prognostic predictor. Plasma samples were collected from a total of 33 ovarian cancer patients prior to and after primary treatment including surgery and chemotherapy. CA125 returned to normal plasma concentrations within the first month of treatment in almost all patients even in the cases with unresectable tumor, whereas there was a remarkable mix of variation in the concentrations of plasma miR-200b. The patients with a negative miR-200b variation had a longer PFS than those patients with a positive variation, and the risk of disease-progression was significantly higher in patients with a positive variation of miR-200b compared to those with a negative variation [80]. Benson et al. conducted a pilot study to identify plasma miRNAs that can predict outcomes of a specific chemotherapy regimen: carboplatin with decitabine, a hypomethylating agent. Plasma samples were collected from 14 recurrent platinum-resistant ovarian cancer patients prior to and at the end of the first cycle of treatment, and the changes in plasma miRNA concentrations were evaluated. Lower concentrations of circulating plasma miR-148b-5p were associated with worse PFS, suggesting its potential as a novel biomarker of therapeutic response [84].

## Can circulating miRNA predict the response to novel cancer treatments?

While a variety of molecular targeted therapies (e.g. bevacizumab, olaparib, or cediranib (a pan-VEGFR inhibitor) [85] have been developed for ovarian cancer treatment and showed some improvements in progression-free survival of patients, no significant benefits with respect to overall survival have been reported so far [7–10, 85]. This is partly due to the lack of methods to discriminate between patients who will or will not benefit from these novel treatments. Since these therapies would be applied mainly for relapsed ovarian cancer patients and it would not be practically possible to obtain tumors in most relapsed cases, the identification of novel biomarkers from body fluids such as serum, plasma, whole blood, and urine would be indispensable especially to those patients. Thus, circulating cell free miRNAs in body fluids are to be good candidates and need to be extensively analyzed. At present, there have been no reports which showed the association between these novel drugs and circulating miRNAs in ovarian cancer; however, the recent study in metastatic colorectal cancers revealed that changes in circulating plasma miR-126 during treatment were predictive of tumor response to the first-line chemotherapy (XELOX; capecitabine and oxaliplatin) combined with bevacizumab [86]. While the responding patients had a significant decrease of circulating miR-126, this circulating miRNA modestly increased in non-responding patients. MiR-126 is known to have a high endothelial cell (EC) specificity and involved in EC proliferation, migration, survival, and in the regulation of blood vessel integrity [87, 88], indicating that the circulating level of this miRNA may reflect the response to bevacizumab. Such efforts would be taken in ovarian cancer treatment in the near future.

## Can circulating miRNAs be therapeutic targets for cancer treatment?

He WA, et al. reported cancer-secreted microvesicles contain an elevated expression of miR-21 and induce myoblast apoptosis, which lead to cancer cachexia of patients *via* a Toll-like receptor 7 (TLR7) [89]. They provided insights into therapeutic avenues for cachexia, possibly by inhibiting microvesicles secretion, inhibiting fusion of microvesicles with muscle cells, or blocking the binding of miR-21 to TLR7/8. Ciravolo et al. reported that the exosomes released by the HER2-overexpressing breast cancer cell lines express a full-length HER2 molecule and that these exosomes bound to trastuzumab and inhibited its anticancer cell proliferative activity [90]. Based on this preclinical data, Aethlon Medical Inc. (CA, USA) has developed HER2osome™, as a therapeutic strategy to combat HER2 positive breast cancer through the capture of circulating HER2-positive exosomes [91]. Although promising results from these novel devices in the clinical settings have not been reported so far, such approaches to eliminate circulating microvesicles containing miRNAs have the potential to be a breakthrough in cancer therapy.

## Conclusions

In recent years, emerging evidence has suggested that circulating miRNAs may hold great potential as promising biomarkers for early detection, prognosis, and sensitivity to chemotherapy of ovarian cancer. However, to date, most of the studies appear to be preliminary, because they simply identified altered levels of circulating miRNAs in ovarian cancer patients with relatively small cohort sizes. They lack direct comparison or in combination with conventional diagnostic procedures, such as CA125 and ultrasonography, particular for early stage diseases. In addition, the lack of standardized protocols including sample collection, RNA extraction, and the selection of suitable internal control makes it difficult to compare the results between studies reported. There have been inconsistent findings about circulating miRNAs from the same tumor reported by different studies. Nevertheless, circulating miRNAs have potential as novel non-invasive and highly useful biomarkers of ovarian cancer, as shown in various types of disease such as cardiovascular disease, diabetes mellitus, and cancer of other organs [92]. Further studies with standardized procedures and at larger scales are warranted to enhance the consideration of the clinical significance of circulating miRNAs in ovarian cancer. Recently several big projects focusing on circulating miRNAs as a biomarker have launched. For instance, NIH launched the Extracellular RNA Communication program to advance the field of extracellular miRNA research in 2013. The NIH Common Fund awarded approximately $130 million to 30 research projects to investigate the diagnostic and therapeutic potential of circulating miRNAs [93]. In Japan, a big project led by the New Energy and Industrial Technology Development Organization (NEDO), a national research agency, started in 2014. This project aims to enable the early detection of 13 types of cancers including ovarian cancer using circulating miRNAs from blood samples of 65,000 people [94]. These projects may be great milestones to establish miRNA-based ovarian cancer detection. In addition, several basic research studies have demonstrated that circulating miRNAs can be mediators of cell-to-cell communication during cancer progression [89, 95, 96]. There is still a long way, however, and further evidence would make it possible to use circulating miRNAs not only as biomarkers but also as potential therapeutic targets in the future.
